# Navigating challenges and shaping futures through mental health nursing in the United Kingdom

**DOI:** 10.1111/jpm.13092

**Published:** 2024-08-07

**Authors:** Dean McShane, Amy Madden, Lucy Kinsey, Oladayo Bifarin

**Affiliations:** ^1^ School of Nursing and Advanced Practice, Faculty of Health Liverpool John Moores University Liverpool UK; ^2^ Mersey Care NHS FT Liverpool UK

**Keywords:** mental health, nursing, registered mental health nurse

## Abstract

**What is known on the subject?:**

- Mental health nurses in the UK play a critical role in providing care, advocating for patients and navigating the complexities associated with mental health challenges.
- Acknowledging and supporting the efforts of mental health nurses is essential for advancing mental health care, promoting inclusivity and fostering community resilience amidst ongoing challenges within existing health care organisations.

**What this paper adds to existing knowledge?:**

- Highlights the integral role of mental health nurses in the UK's health system as both care providers and advocates, underpinning the importance of addressing the growing demand for mental health services
- Introduces innovative digital tools like the ‘Hub of Hope’ and the potential of social prescribing as effective strategies for improving access to mental health care and support.
- Emphasises the need for enhanced support and recognition of mental health nurses to ensure the sustainable advancement of mental health services and the well‐being of communities.

**What are the implications for practice?:**

- Mental health nurses should integrate innovative digital tools like the ‘Hub of Hope’ into their practice to improve signposting and access to mental health resources for individuals in need.
- There is a critical need for ongoing education and support for mental health nurses within practice settings to maintain their well‐being and enhance their ability to provide holistic care despite increasing clinical demands.
- Advocacy for increased investment in mental health resources and support for research activities lead by mental health nurses is essential to address current service gaps and promote the development of more effective interventions.

1

**Key points**

This article showcases the crucial role played by mental health nurses (RMNs) in the UK, focusing on their significant efforts to mitigate existing mental health inequalities pathways. It is important in terms of adopting new digital technologies and social prescribing as essential methods to improve access to care and bridge the gaps in treatment. We specifically illustrate the vital role mental health nurses play in advocating for patients, coordinating care, and introducing innovative solutions in the face of ongoing obstacles related to access and the distribution of resources. As a result, it highlights the pressing need for enhanced support and investment in mental health services to improve care delivery, close gaps in services, and promote community health.

## 2 BACKGROUND

Each year International Nurses Day highlights the role of the registered nurse. This papers aim is to highlight the role that mental health nurses (MHNs) play in promoting mental health for all, protecting those at risk, and advancing mental health care in the community (WHO, 2022). Registered Nurses specialising in mental health are biopsychosocial specialists who support individuals with a range of mental health challenges through their clinical and personal recoveries (Gournay, 2021). Specifically, these nurses provide comprehensive care designed to either facilitate recovery from illness or enable individuals to lead fulfilling lives, especially when they are coping with the continuous symptoms of a chronic mental illness (National Careers Service, 2019; NHS, 2019). Making up 44% of the entire mental health nursing workforce, MHNs are expected to advocate for patients; challenge stigma and discrimination, coordinate care, provide leadership, educate both patients and colleagues to understand and manage mental health conditions effectively, and actively participate in delivering or implementing various research activities (International Council of Nurses, 2024). A central aspect of the role of mental health nurses is to develop and maintain therapeutic relationships with those affected by mental health difficulties—including patients, carers and health and social care professionals—throughout the nursing process (Bifarin, 2016). Therefore, mental health nurses are expected to employ a range of strategies, from socialisation to positive behaviour activation, while supporting patients. This approach aims to empower individuals living with mental health difficulties to have control over their health (Delaney et al., 2018).

A career in mental health nursing offers unique opportunities to make a difference, providing flexibility and promising employment prospects (NHS, 2019). The distinct and multidimensional focus of their role is essential in overcoming community and structural barriers that hinder a comprehensive understanding of mental health as a complex continuum. MHNs' multifaceted responsibilities make them indispensable in providing comprehensive mental health care as they fulfil various roles including counselling, crisis intervention and case management (Stewart, Naegle, et al., 2024).

They play a vital role in assisting mental health care systems in building resilience and their insights and advocacy help shape policies that support mental health care improvements for inclusive, equitable and accessible mental health services for all (Stewart, Naegle, et al., 2024).

These efforts are particularly crucial for vulnerable populations who might otherwise face significant barriers to accessing mental health services. Despite the significant contributions of mental health nurses, resources for mental health care remain limited and unevenly distributed across many countries, with a disproportionately small segment of health budgets dedicated to mental health (WHO, 2022). As a result, disparities in mental health care continue to widen, rendering access increasingly unattainable for many and exacerbating the treatment gap for severe mental health conditions, which in some regions, reaches as high as 90% (WHO, 2022). Given the effects of globalisation, social and economic pressures, prolonged conflicts and public health crises worldwide, the crucial role of mental health nurses in care delivery must be recognised and valued.

In the United Kingdom, the mental health services are experiencing some of the most significant staff shortages within the National Health Service (Adams et al., 2021), and are facing challenges, with more than 1.8 million people currently on waiting lists, highlighting the overstretched and understaffed conditions of these services (Deakin, 2023). A health report in Scotland (Health and Social Care Alliance Scotland, 2017) suggests a substantial number of people seeking health services report mental health‐related issues. However, the Scottish Action for Mental Health (2017) report, ‘Know Where to Go,’ highlighted that approximately 800,000 adults lacked knowledge on accessing help. Likewise, in England, Tunks et al. (2023) identified various barriers to accessing support for common mental health difficulties across different populations, including challenges related to knowledge, attitudes associated with stigma, systems and relationships, from early symptom detection to interactions with primary care services. Furthermore, recent anti‐stigma campaigns and increased public awareness have significantly shifted perceptions and debunked myths surrounding mental health difficulties within the UK, leading to greater likelihood of individuals seeking support and help (Henderson et al., 2020; Rethink, 2023). Nevertheless, MHNs are in precarious positions with adverse effects on their specialist roles as an increasing number of individuals seeking help for mental health issues are not receiving support. For instance, it is estimated that two‐thirds of those who die by suicide are not engaged with NHS mental health services (Healthcare Quality Improvement Partnership, 2018), and the past decade has seen a concerning rise in suicide rates (Office For National Statistics, 2021).

NHS organisations express deep concerns over the escalating unmet needs, particularly among children and young people (NHS Providers, 2023). Yet, considering the lengthy NHS mental health services waiting times, it has become crucial for those working in mental health, especially nurses, to possess and utilise effective signposting skills within the community (Stacey‐Emile, 2020). When General Practitioners (GPs) were surveyed about the type of mental health information that would be beneficial, 87.3% indicated a preference for guides on local resources, including community activities like walking groups, stress management groups and voluntary sector support services (Scottish Government, 2015). Also, research by ‌Saxild et al. (2023) and Thomas et al. (2016) indicates that guiding individuals to digital and practical online resources can be an effective strategy for managing various mental health conditions. Following this background, ‘Mental Health Promotion and Prevention’ will highlight some of the innovative strategies being used to address the increasing demand for mental health care services. This paper will introduce digital tools that are effective for improving access to services. In ‘Leadership’, we articulate implications for practice through sharing an evidence‐based pathway for addressing some current gaps in treatment/service and showing the importance of leadership and structure in ensuring well‐being for patients and healthcare professionals. Lastly, ‘Increasing Research Capacity in Mental Health’ shows how lack of investment in mental health limits the ability or capacity of MHNs to support their patients with managing mental health effectively within the context of nurses recognising the importance of participating in research that will contribute to identifying innovative interventions for enhanced access to care. The following sections focus on mental health promotion and prevention: finding and accessing mental health services.

Access to a wide range of interventions for mental health and well‐being is available within local communities in the UK. These include mental health sports programs (e.g. football therapy, fishing the mind and park run), peer‐led self‐support groups (e.g. Andy's Man Club and Men Sheds), support from mental health charities (e.g. James's place, mind and various recovery colleges or well‐being campuses) and modern technologies like apps and podcasts that are mental health focused. Mental health nurses' familiarity with available services, charities or groups within their locality is invaluable for advancing public health, reducing stigma, protecting human rights and supporting development. For instance, mental health signposting apps like the ‘ *Hub of Hope* ’, play a crucial role in locating local mental health charities and organisations to provide direct support to individuals in need. The Hub of Hope boasts a comprehensive database of over 13,000 mental health services, including 24/7 crisis support from Samaritans and the Shout text service (Central and Northwest London NHS Foundation Trust, 2023). It has rapidly become the UK's fastest growing and most comprehensive mental health support database, freely accessible at any time. The *Hub of Hope* is widely utilised by numerous organisations, including Samaritans, NHS England and various ambulance services, to effectively guide people to appropriate support. Developed by the national charity *Chasing the Stigma*, the Hub of Hope significantly stands as the UK's leading mental health support database, amalgamating local, national, peer, community, charity, private and NHS mental health support services for the first time, with the aim of directing individuals towards essential mental health support in their vicinity. Within, nurse education, digital technologies are leveraged to ensure that all nursing students receive training in signposting with a particular emphasis on addressing stigma from their first year, including a mental health session on how to utilise the Hub of Hope. Feedback from students has been overwhelmingly positive, with many noting that the resources available via the platform have enriched their interactions with patients, carers and practitioners during clinical placements.

Another factor to consider when thinking of effective interventions is the importance today's culture places on on‐demand entertainment, as due to its convenience and the vast selection of material available across numerous platforms and providers, the value cannot be overstated (Casares, 2022). Podcasts, compatible with today's entertainment landscape, have witnessed a significant surge in production volume and popularity over the last 5 years (Kachka, 2019). Riddell et al. (2020) explored the listening behaviours of educational podcast users, finding them simple to use and entertaining, facilitating focused study and broad content exposure. Listening also fostered a sense of connection among participants with their peers, supervisors and the wider professional community. Accordingly, educational podcasts are increasingly recognised as an effective medium for teaching and learning within health professions education. An example of this is ‘ *The Man Hug* ’, a podcast hosted by four nurse academics, which focuses on men's mental health, employing lived experience storytelling and signposting to educate its audience about mental health services available in their locality. The format typically includes a main guest sharing their story, highlighting current statistics related to the theme to normalise the conversations, and promoting local services and organisations to enhance signposting knowledge. In its first year in 2023, an evaluation of the podcast by mental health student nurses provided insights into its acceptability as an educational tool. From 36 responses, 92% affirmed that the podcast had broadened their awareness of existing services within their locality for referrals and enhanced their signposting knowledge, and 100% felt confident in using the ‘*Hub of Hope*’ app with individuals in the future.

In this section, we focus on mental health nurse leadership, supervision and education in the context of a self‐harm pathway. Aside from accessing mental health support by digital or online resources, the self‐harm pathway represents an evidence‐based care model within inpatient settings. This was designed for individuals who engage in self‐harm within inpatient settings. Originating in 2014 through a collaborative effort by an inpatient nursing team and psychologists in the Northwest of England, this pathway is implemented by a multidisciplinary team (MDT) dedicated to teaching skills that enable patients to understand and manage self‐harm effectively. These skills are conveyed through structured, individualised sessions, enhancing patient care and support (Parker et al., 2022). Addressing self‐harm in patients can be emotionally taxing, often negatively impacting the well‐being of the staff involved (O'Connor & Glover, 2017). Thus, the sustainability and effectiveness of the pathway are heavily reliant on the provision of quality structured supervision. Such supervision serves as a supportive mechanism, fostering reflection within the MDT and promoting a culture of continuous learning and support (Howard & Eddy‐Imishue, 2020). Supervision is systematically integrated into the managerial supervision schedules and is a focal point of regular reflective practice meetings on the ward. Through consistent supervision, the inpatient team can effectively monitor shifts in cultural attitudes towards the management of self‐harm and work towards minimising the likelihood of discrimination potentially resulting in restrictive practices. This structured approach to supervision and education underlines the importance of leadership in ensuring the pathway's effectiveness and the overall well‐being of patients and staff. Central to the pathway's success is a focus on staff education, which equips the MDT with the knowledge and tools to respond to self‐harm incidents effectively, safely and in the least restrictive manner possible (Care Quality Commission, 2015). Education and training are facilitated by a consultant clinical psychologist and members of the inpatient workforce who possess clinical experience with the pathway. This approach fosters a collaborative learning environment, where best practices, practical examples and innovative strategies are shared among practitioners. Specifically, education is delivered through evidence‐based teaching sessions within divisions, emphasising the pathway's implementation and delivery as a core component of the induction process for staff on female acute inpatient wards.

This section focuses on the need for increasing research capacity in mental health care and interventions. One of the unintended consequences stemming from the underinvestment in mental health is the limited research capacity and capability, particularly when juxtaposed with physical health research (Woelbert et al., 2019). This situation contradicts the objectives outlined in the Comprehensive Mental Health Action Plan 2013–2030, which envisions a world where mental health is prioritised, focusing specifically on enabling individuals affected by various mental health difficulties to exercise their fundamental human rights, access high‐quality care and participate in society without facing stigma and discrimination (WHO, 2021). In pursuit of evidence‐based mental health care—characterised by precision, proof of effectiveness and personalisation—mental health nurses are increasingly recognising the importance of actively engaging in research activities. These activities range from consulting with marginalised groups and driving Public and Patient Involvement Engagements (PPIE) to leading and supporting others in seizing research opportunities such as grant writing and internships.

Thanks to funding from the Office of Life Science and the National Institute for Health and Care Research, the Mental Health Research for Innovation Centre (M‐RIC) is initiating efforts to address inequalities in regions of the UK that have been overlooked. Mental health nurses are at the forefront of devising innovative strategies to foster inclusivity in mental health research, ensuring that all patients and staff, regardless of their backgrounds, are informed about opportunities to participate in research. The investment in mental health research is critical for enhancing access to care and developing appropriate interventions. These interventions are vital for addressing the immediate need to overhaul and expand mental health care services for a significant impact within various communities (WHO, 2022). To maintain and amplify this impact, there is a pressing need to develop human resources in terms of capacity. Such development is crucial for working closely with individuals affected by mental health issues and establishing timely connections with community stakeholders. Subsequently, the demand for nurses is expected to create 30 million new positions, especially in low‐ to middle‐income countries, and with women performing 76% of unpaid care work, often in low‐wage roles, investing in nursing can help close these gender gaps by improving pay, working conditions and career advancement opportunities (Stewart, Adynski, et al., 2024). This can be empowering by providing workers with decent jobs as this growth in nursing employment contrasts with declines in other sectors and offers a pathway out of poverty for women and girls. Ultimately, better health through improved nursing care can add up to USD 12 trillion to global GDP by 2040, translating to 0.4% faster growth each year and better mental health means an increase in workforce participation and productivity (Stewart, Adynski, et al., 2024).

In conclusion, the evolving landscape of mental health nursing within the UK underscores the critical role of MHNs in addressing the complex challenges faced by individuals with mental health conditions. Amidst increasing demands for mental health services and persistent disparities in care access, MHNs are at the forefront of pioneering innovative approaches to care, education and research. Their dedication to patient advocacy, interdisciplinary collaboration and continuous professional development is instrumental in bridging the gap between mental health needs and the provision of effective, compassionate care. Nursing is a crucial area for policy and financial support, as in addition to improving health outcomes, it drives economic growth, gender equality and social well‐being. making it a critical area for policy and financial support. As the mental health sector navigates through resource limitations and societal stigma, the commitment of MHNs to advancing mental health care, promoting inclusivity and fostering resilience within communities remains indispensable. Recognising and supporting the vital contributions of these professionals is essential for the sustainable enhancement of mental health services and the realisation of a more equitable healthcare landscape.

## FUNDING INFORMATION

There is no financial interest to report.

## CONFLICT OF INTEREST STATEMENT

Oladayo Bifarin is a National Institute for Health and Care Research Leader. The views expressed in this article are those of the author(s) and not necessarily those of NIHR or the Department of Health and Social Care.

## Data Availability

Data sharing is not applicable to this article as no new data were created or analyzed in this study.

## References

[jpm13092-bib-0001] ‌Adams, R. , Ryan, T. , & Wood, E. (2021). Understanding the factors that affect retention within the mental health nursing workforce: A systematic review and thematic synthesis. International Journal of Mental Health Nursing, 30(6), 1476–1497. 34184394 10.1111/inm.12904

[jpm13092-bib-0002] Bifarin, O. (2016). Professional socialisation: The art in mental health nursing practice. British Journal of Mental Health Nursing, 5(5), 228–231.

[jpm13092-bib-0003] Care Quality Commission . (2015). Mental Health Act Code of Practice . https://www.cqc.org.uk/sites/default/files/20190625_mhacop‐report.pdf

[jpm13092-bib-0004] Casares, D. R. (2022). Embracing the podcast era: Trends, opportunities, & implications for counsellors. Journal of Creativity in Mental Health, 17(1), 123–138.

[jpm13092-bib-0005] Central and Northwest London NHS Foundation Trust . (2023). Signposting to hope this winter – The Hub of Hope app: Central and North‐West London NHS Foundation Trust . https://www.cnwl.nhs.uk/news/signposting‐hope‐winter‐hub‐hope‐app

[jpm13092-bib-0201] Deakin (2023). https://www.england.nhs.uk/2023/01/major‐plan‐to‐recover‐urgent‐and‐emergency‐care‐services/

[jpm13092-bib-0007] Delaney, K. R. , Naegle, M. A. , Valentine, N. M. , Antai‐Otong, D. , Groh, C. J. , & Brennaman, L. (2018). The effective use of psychiatric mental health nurses in integrated care: Policy implications for increasing quality and access to care. The Journal of Behavioral Health Services & Research, 45, 300–309. 28484943 10.1007/s11414-017-9555-x

[jpm13092-bib-0008] Gournay, S. (2021). ‘*As mental health nurses, we are biopsychosocial specialists*’. [online] nursing times. https://www.nursingtimes.net/opinion/as‐mental‐health‐nurses‐we‐are‐biopsychosocial‐specialists‐05‐10‐2021/

[jpm13092-bib-0009] Health and Social Care Alliance Scotland . (2017). https://www.alliance‐scotland.org.uk/wp‐content/uploads/2017/10/ALLIANCE‐Developing‐a‐Culture‐of‐Health.pdf

[jpm13092-bib-0010] Healthcare Quality Improvement Partnership . (2018). The assessment of clinical risk in mental health services National Confidential Inquiry into Suicide and Safety in Mental Health . https://documents.manchester.ac.uk/display.aspx?DocID=38466

[jpm13092-bib-0011] Henderson, C. , Potts, L. , & Robinson, E. J. (2020). Mental illness stigma after a decade of time to change England: Inequalities as targets for further improvement. European Journal of Public Health, 30(3), 497–503. 10.1093/eurpub/ckaa013 PMC7292343 32531039

[jpm13092-bib-0012] Howard, V. , & Eddy‐Imishue, G. E. K. (2020). Factors influencing adequate and effective clinical supervision for inpatient mental health nurses' personal and professional development: An integrative review. Journal of Psychiatric and Mental Health Nursing, 27(5), 640–656. 31981445 10.1111/jpm.12604

[jpm13092-bib-0013] International Council of Nurses . (2024). Mental Health Nursing Guidelines . https://www.icn.ch/sites/default/files/2024‐03/ICN_MentalHealthNursingGuidelines‐2024_FINAL_EN_0.pdf

[jpm13092-bib-0014] Kachka, B. (2019). Are we in a podcast bubble? Podcasting is suddenly more than a mission; it's an industry. *Vulture* . https://www.vulture.com/2019/03/are‐we‐in‐a‐podcast‐bubble.html

[jpm13092-bib-0015] National Careers Service . (2019). *Mental health nurse* | *Explore careers*. [online]. https://nationalcareers.service.gov.uk/job‐profiles/mental‐health‐nurse

[jpm13092-bib-0016] NHS . (2019). Mental Health Nurse. [online] Health Careers . https://www.healthcareers.nhs.uk/explore‐roles/nursing/roles‐nursing/mental‐health‐nurse

[jpm13092-bib-0017] NHS Providers . (2023). *NHS Providers responds to call for long term mental health plan*. [online]. https://nhsproviders.org/news‐blogs/news/nhs‐providers‐responds‐to‐call‐for‐long‐term‐mental‐health‐plan

[jpm13092-bib-0018] O'Connor, S. , & Glover, L. (2017). Hospital staff experiences of their relationships with adults who self‐harm: A meta‐synthesis. Psychology and Psychotherapy: Theory, Research and Practice, 90(3), 480–501. 10.1111/papt.12113 28035740

[jpm13092-bib-0019] Office For National Statistics . (2021). *Suicides in England and Wales ‐ Office for National Statistics*. [online]. https://www.ons.gov.uk/peoplepopulationandcommunity/birthsdeathsandmarriages/deaths/bulletins/suicidesintheunitedkingdom/2020registrations

[jpm13092-bib-0020] Parker, B. , Swift, E. , & Gkika, S. (2022). Staff and service users' experiences of the self‐harm pathway on an acute inpatient ward. British Journal of Mental Health Nursing, 11(2), 1–10.

[jpm13092-bib-0021] Rethink . (2023). *Stigma and discrimination*. [online]. https://www.rethink.org/campaigns‐and‐policy/policy‐and‐influencing/stigma‐and‐discrimination/

[jpm13092-bib-0022] Riddell, J. , Robins, L. , Brown, A. , Sherbino, J. , Lin, M. , & Ilgen, J. S. (2020). Independent and interwoven: A qualitative exploration of residents' experiences with educational podcasts. Academic Medicine, 95(1), 89–96. 31517682 10.1097/ACM.0000000000002984

[jpm13092-bib-0023] ‌Saxild, S. , Wilson, P. , de Voss, S. , & Overbeck, G. (2023). Clinicians' experiences in signposting an online mental health resource to expectant mothers: A qualitative study. BMC Pregnancy and Childbirth, 23(1), 336. 37165318 10.1186/s12884-023-05671-w PMC10173643

[jpm13092-bib-0024] Scottish Action for Mental Health . (2017). https://www.samh.org.uk/documents/samh_know_where_to_go_‐_your_guide.pdf

[jpm13092-bib-0025] Scottish Government . (2015). Main Report of the National Review of Primary Care Out of Hours Services . https://www.gov.scot/publications/main‐report‐national‐review‐primary‐care‐out‐hours‐services/pages/17/

[jpm13092-bib-0026] Stacey‐Emile, G. (2020). Advocating mental health promotion. In Promoting Health and Wellbeing: For nursing and healthcare students (p. 121). Lantern Publishing Ltd.

[jpm13092-bib-0027] Stewart, D. , Adynski, G. , Bertoni, K. , Catton, H. , Chopra, M. , Contandriopoulos, D. , Duckett, S. , Lafortune, G. , Lauer, J. , McClelland, A. , Needleman, J. , Parish, C. , Shannon, G. , Spetz, J. , Thompson, R. , & Wagner, L. (2024). International Nurses Day 2024: The economic power of care. International Council of Nurses. https://www.icn.ch/sites/default/files/202405/ICN_IND2024_report_EN_A4_6.1_0.pdf

[jpm13092-bib-0028] Stewart, D. , Naegle, M. , Rolland, E. , Hughes, F. , & Ryan, K. (2024). Geneva . https://www.icn.ch/sites/default/files/2024‐03/ICN_MentalHealthNursingGuidelines‐2024_FINAL_EN_0.pdf

[jpm13092-bib-0029] Thomas, S. , Jenkins, R. , Burch, T. , Calamos Nasir, L. , Fisher, B. , Giotaki, G. , Gnani, S. , Hertel, L. , Marks, M. , Mathers, N. , & Millington‐Sanders, C. (2016). Promoting mental health and preventing mental illness in general practice. London Journal of Primary Care, 8(1), 3–9. 10.1080/17571472.2015.1135659 PMC5330334 28250821

[jpm13092-bib-0202] Tunks, A. , Berry, C. , Strauss, C. , Nyikavaranda, P. , & Ford, E. (2023). Patients’ perspectives of barriers and facilitators to accessing support through primary care for common mental health problems in England: A systematic review. Journal of Affective Disorders, 338, 329–340. 37348656 10.1016/j.jad.2023.06.035

[jpm13092-bib-0030] Woelbert, E. , Kirtley, A. , Balmer, N. , & Dix, S. (2019). How much is spent on mental health research: Developing a system for categorising grant funding in the UK. The Lancet Psychiatry, 6(5), 445–452. 30824371 10.1016/S2215-0366(19)30033-1

[jpm13092-bib-0031] World Health Organisation . (2022). *Transforming mental health for all*. [online]. https://iris.who.int/bitstream/handle/10665/356119/9789240049338‐eng.pdf?sequence=1

[jpm13092-bib-0032] World Health Organization . (2021). *Comprehensive Mental Health Action Plan 2013–2030*. [online]. https://www.who.int/publications/i/item/9789240031029

